# Methicillin-Sensitive Staphylococcus aureus Co-infection in Adolescent Patients With Delta-Variant SARS-CoV-2 Acute Respiratory Failure

**DOI:** 10.7759/cureus.52164

**Published:** 2024-01-12

**Authors:** Courtney M Cox, Mireille Liboiron, Heather L Young, Sanjiv Pasala, Matthew P Malone

**Affiliations:** 1 Pediatric Critical Care Medicine, University of Arkansas for Medical Sciences, Little Rock, USA; 2 Pediatric Infectious Diseases, University of Arkansas for Medical Sciences, Little Rock, USA

**Keywords:** va ecmo, extracorporeal membrane oxygenation (ecmo), pediatric intensive care unit (picu), covid-19, pediatric ards, immune modulation therapy, vv ecmo

## Abstract

We report a series of five pediatric patients admitted with acute respiratory failure due to delta-variant SARS-CoV-2, found to have a methicillin-sensitive *Staphylococcus aureus* (MSSA) co-infection. All five patients required escalation of their respiratory support within 24 hours of discovering the MSSA infections. Four out of the five patients received immune-modulating therapies. Four patients required extracorporeal membrane oxygenation support. One patient died, and the other four survived until hospital discharge. Clinicians should consider secondary bacterial infections in patients with COVID-19 treated with immune modulators. MSSA co-infection can lead to increased morbidity and mortality in patients with COVID-19.

## Introduction

SARS-CoV-2 is a novel coronavirus first reported in December 2019 in Wuhan, China [1]. COVID-19 is the name given to the clinical symptomatology caused by SARS-CoV-2, which can involve fever, cough, and myalgias, among other clinical symptoms [2]. Initially, it was reported that COVID-19 caused a generally mild disease in children, with few requiring hospitalization [3-5]. As of November 2023 in Arkansas, USA, children aged 17 years and younger accounted for approximately 200,000 cases with over 900 hospitalizations, with 50 deaths in individuals younger than 24 [6]. Comparatively, there have been over 15 million cases nationally in children under the age of 17 years, with 1,800 deaths [7].

Bacterial, viral, and fungal co-infections are known complications of viral respiratory illnesses. Bacterial co-infections are associated with increased mortality [8]. There have been multiple reports in adults on the incidence and type of co-infections seen in COVID-19, but few in pediatric patients. Adult data show that co-infections are present in approximately 13-45% of patients with COVID-19, most commonly with the bacterial pathogens *Mycoplasma pneumoniae* and *Haemophilus influenzae* [8-14]. Bacterial co-infection rates are higher in critically ill patients [4]. Viral co-infections are also reported, with the most common pathogens being the influenza virus, rhinovirus/enterovirus, and respiratory syncytial virus [8-14].

Our report aims to describe a series of adolescent patients admitted to our pediatric intensive care unit with acute respiratory failure due to delta-variant SARS-CoV-2, found to have a methicillin-sensitive *Staphylococcus aureus* (MSSA) co-infection. We describe five patients with acute delta-variant SARS-CoV-2 infection, requiring intubation and mechanical ventilation, all of whom had MSSA infections discovered within 24 hours of escalating respiratory support. This case series was determined to be exempt by the Institutional Review Board at our university.

A portion of this case series was presented as a poster presentation at the 2022 ECMO and the Advanced Therapies for Respiratory Failure Symposium on February 17, 2022.

## Case presentation

Case 1

**Figure 1:**
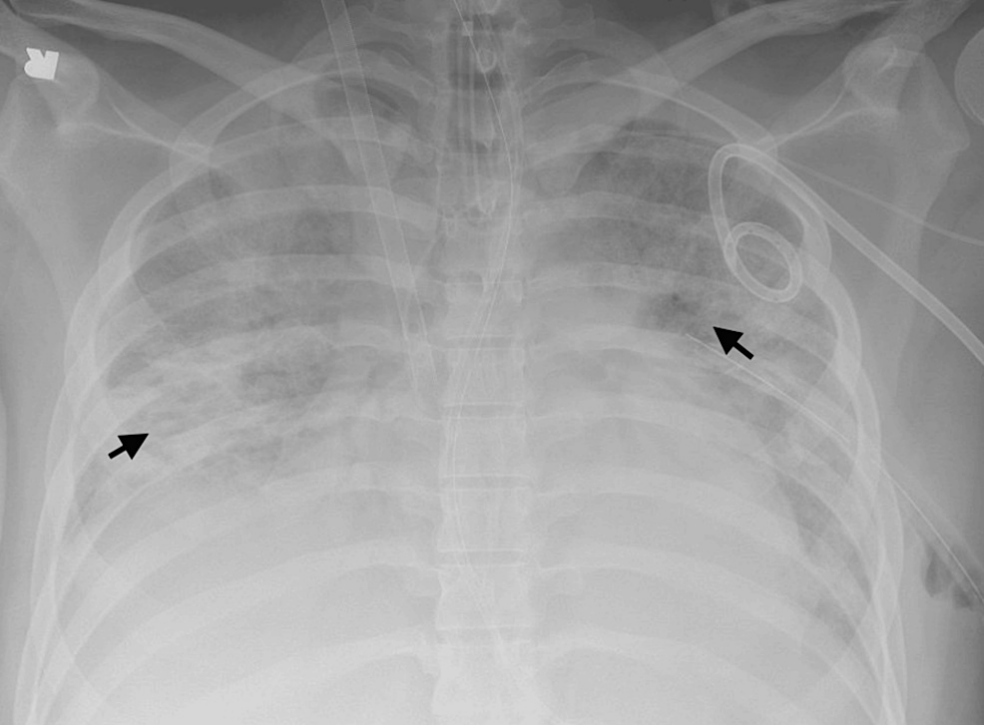
Case 1 chest X-ray Arrows showing bilateral pulmonary infiltrates

A 19-year-old male weighing 116 kg was admitted to a referring hospital with pneumonia due to SARS-CoV-2 on a heated high-flow nasal cannula. He received baricitinib and dexamethasone for COVID-19 treatment. He subsequently developed worsening hypoxemic respiratory failure and required intubation on hospital day 6. He was referred to our facility on hospital day 15 for extracorporeal membrane oxygenation (ECMO) support. MSSA was cultured from a mini-bronchoalveolar lavage (mini-BAL) obtained immediately prior to ECMO cannulation, and all blood cultures were negative. A chest X-ray on the day of ECMO cannulation reveals bilateral pulmonary infiltrates (Figure 1). Pertinent lab values at the time the culture was obtained include the following: CRP 195 mg/L, WBC 8.31 K/uL, and procalcitonin 26.68 ng/mL. He was initially treated with cefepime and vancomycin until cultures resulted in MSSA and subsequently switched to nafcillin. He received a tracheostomy while on ECMO and had a 48.5-day ECMO run. He received intensive inpatient rehab and was decannulated from his tracheostomy prior to hospital discharge.

Case 2

**Figure 2:**
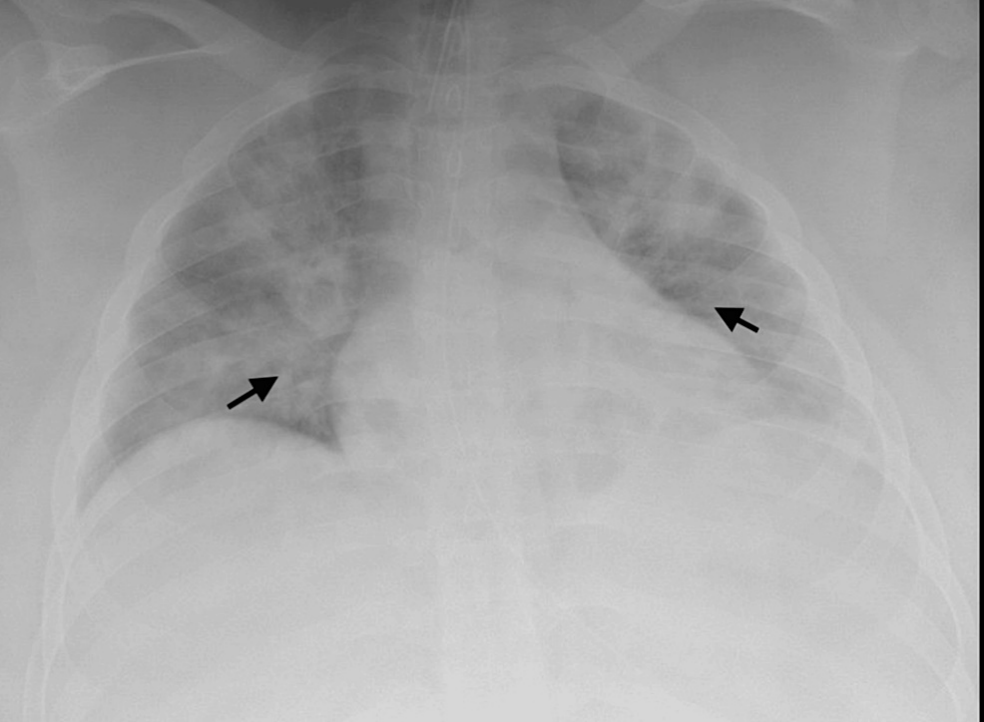
Case 2 chest X-ray Arrows indicating bilateral pulmonary infiltrates

A 16-year-old male weighing 118 kg was admitted with pneumonia due to SARS-CoV-2 on a heated high-flow nasal cannula. He received remdesivir, tociluzimab, and dexamethasone for COVID-19 treatment. He required intubation on hospital day one for progressive hypoxemia. MSSA was cultured from a mini-BAL obtained immediately after endotracheal intubation. The chest X-ray at the time of intubation showed worsening bilateral infiltrates (Figure 2). Pertinent lab values at the time the culture was obtained include the following: WBC count 4.44 K/uL, CRP 36 mg/L, and procalcitonin 0.07 ng/ML. He was initially treated with ceftriaxone until cultures resulted in MSSA and subsequently switched to nafcillin. His respiratory failure progressed, and he developed acute respiratory distress syndrome (ARDS), requiring venovenous (V-V) ECMO 14 days after intubation. He subsequently suffered a cardiac arrest and was converted to venoarteriovenous (VA-V) ECMO. He remained on ECMO support for 25 days and had a post-ICU complication of pulmonary emboli, despite prophylactic enoxaparin. He still struggles with exercise-induced shortness of breath and follows with pulmonology closely.

Case 3

**Figure 3:**
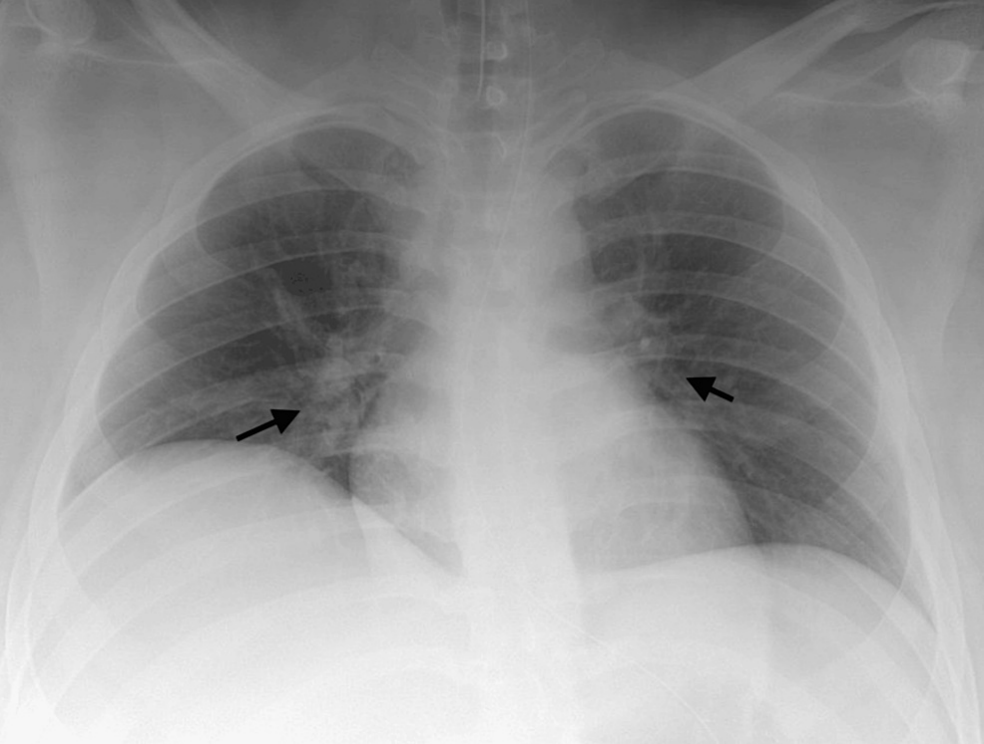
Case 3 chest X-ray Arrows indicating bilateral pulmonary infiltrates

A 17-year-old male weighing 107 kg was admitted for hyperosmolar hyperglycemic syndrome, presumed to be secondary to an acute SARS-CoV-2 infection. He had no respiratory symptoms upon presentation but quickly developed an altered mental status, requiring endotracheal intubation on hospital day one. MSSA was cultured from a mini-BAL obtained immediately after intubation. The chest x-ray showed diffuse opacification of each hemithorax (Figure 3). Pertinent lab values at the time the culture was obtained include the following: WBC 12.52 K/uL and CRP 99 mg/L. He was treated with ceftriaxone, vancomycin, and metronidazole due to the severity of his illness. After intubation, he developed progressive hypotension and shock, requiring multiple vasopressors and worsening hypoxemia. He was placed on V-V ECMO support on hospital day 2 and converted to VA-V ECMO later that same day after suffering a cardiac arrest. His COVID-19 was treated with tociluzimab and dexamethasone, starting on hospital day two. He required 11 days of ECMO support. He received intensive inpatient rehabilitation and still receives outpatient therapy for his critical illness, neuropathy.

Case 4

A 16-year-old male with trisomy 21 weighing 54 kg was admitted with pneumonia due to SARS-CoV-2 on a heated high-flow nasal cannula but was quickly transitioned to non-invasive mechanical ventilation one hour after admission. He received dexamethasone and remdesivir for COVID-19. He remained on non-invasive mechanical ventilation until hospital day 9, when his hypoxemia worsened and he required endotracheal intubation. Immediately after intubation, he suffered a cardiac arrest and was unable to be resuscitated. A blood culture obtained approximately 18 hours prior to intubation was positive for MSSA.

Case 5

**Figure 4:**
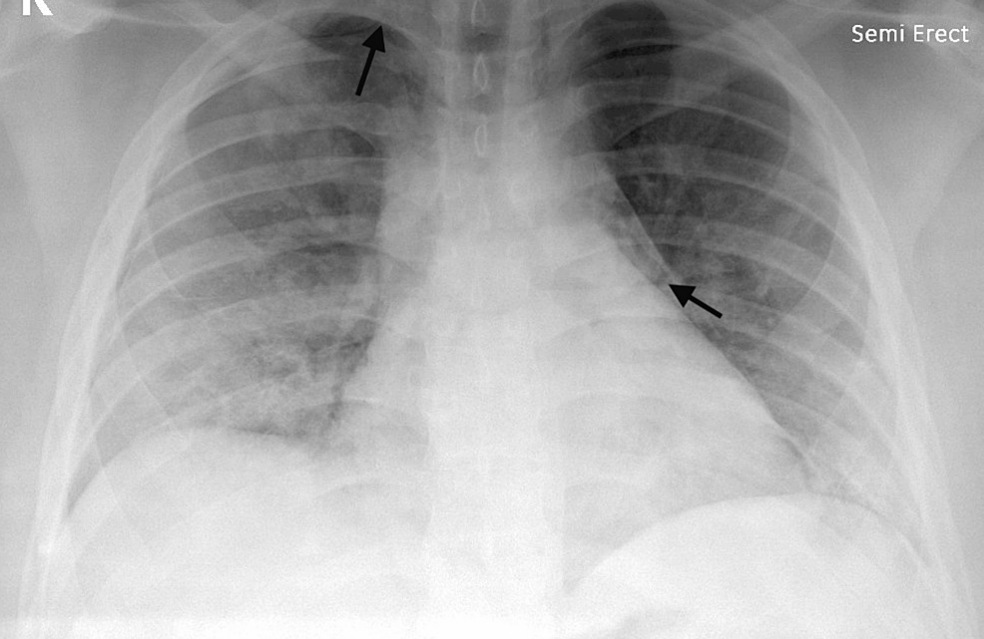
Case 5 chest X-ray Arrows indicating pneumothorax on the right side of the chest and pneumomediastinum on the left side of the chest

A 19-year-old male weighing 114 kg was transferred to our facility on hospital day 2 for worsening pneumonia due to SARS-CoV-2 on a heated high-flow nasal cannula. Initial chest CT obtained at the referring facility showed diffuse groundless and consolidate changes throughout the lungs consistent with known COVID-19 pneumonia. He quickly escalated to non-invasive mechanical ventilation upon admission and subsequently required endotracheal intubation on hospital day 7 for ARDS. He was persistently hypoxemic after intubation, so he was placed on ECMO support. Chest X-rays at that time still showed bilateral pulmonary infiltrates as well as bilateral small apical pneumothoraces and pneumomediastinum (Figure 4). He received dexamethasone and tocilizumab for the treatment of COVID-19. A mini-BAL culture obtained 24 hours after intubation was positive for MSSA, along with a blood culture obtained 72 hours after intubation. Pertinent lab values at the time the culture was obtained include the following: WBC 15.68 K/uL, CRP 11 mg/L, and procalcitonin 0.08 ng/mL. He was initially treated with clindamycin and ceftriaxone but subsequently switched to nafcillin for MSSA. He remained on V-V ECMO for 13 days and was discharged home with a low oxygen requirement. He was lost to follow-up post-discharge.

## Discussion

Our case series describes five adolescent patients with acute delta-variant SARS-CoV-2 respiratory infections who were found to have MSSA infections within 24 hours of escalating respiratory support. Secondary bacterial infections in COVID-19 patients have previously been reported in adults [8-12]. In a recently published study, Pickens et al. reported that 25% of recently intubated adult COVID-19 patients have a bacterial co-infection, of which 79% were either *Streptococcus* species or MSSA [15]. The same paper also reported that there was no link between typical infectious blood biomarkers and BAL positivity [15]. Limited data is available on pediatric patients.

*Staphylococcus aureus* infections are among the most common bacterial infections worldwide. They are responsible for over 100,000 infections in the United States each year and lead to increased morbidity and mortality [16]. In pediatric patients, the incidence of *Staphylococcus aureus* bacteremia is estimated at 20 per 100,000 children per year. *Staphylococcus aureus* infection is associated with severe complications. Early diagnosis and treatment are necessary for successful outcomes [16]. Four of our five patients were diagnosed with respiratory tract MSSA co-infection via a mini-BAL. A recent systematic literature review by Tepper et al. showed that there is a “high degree of sensitivity and specificity of the mini-BAL for diagnosis of pneumonia in ventilated patients,” which shows that the mini-BAL can be used as an acceptable culture medium for the diagnosis of bacterial co-infections in our patients [17].

Four out of the five patients we describe also received immune-modulating therapies with either tocilizumab or baricitinib. Tociluzimab is an anti-IL-6 receptor monoclonal antibody that is thought to mitigate the hyperinflammatory response of severe SARS-CoV-2 infections and reduce the length of mechanical ventilation [18]. Baricitinib is an oral Janus kinase-selective inhibitor used in conjunction with remdesivir for severe COVID-19 and may be more beneficial than remdesivir alone [19]. Both drugs carry the risk of secondary infections due to their immunosuppressive effects [18,19].

We recognize that our case series included patients who were not under the age of 18. At our institution, we admit patients up to age 21 to our pediatric intensive care unit in accordance with the American Academy of Pediatrics Policy Statement on the Age Limits of Pediatrics [20]. We also recognize that all of the patients in this cohort were obese. This small case series does not lend itself to determining a correlation between obesity and MSSA infections in patients with COVID-19 pneumonia. Another limitation is that the CDC national data and Arkansas state data do not have matching age ranges. We included the state data from ages 24 and under to better show the impact on our adolescent population, as described in this case series.

## Conclusions

MSSA co-infection was found in five of our patients with COVID-19 who required intubation for acute respiratory failure. Clinicians should maintain a high index of suspicion and be aware of the possibility of secondary bacterial infections in critically ill COVID-19 patients. Bacterial cultures should be obtained in these patients as clinically indicated. Clinicians should also consider secondary bacterial infections in patients with COVID-19 treated with immune modulators. It is possible that MSSA co-infection can lead to increased morbidity and mortality in patients with COVID-19, as seen in our cohort; however, further studies are needed to prove this association. Clinicians should be vigilant and suspect co-infection when increased respiratory support is required in patients with COVID-19 pneumonia. All of our patients required increased respiratory support within 24 hours of discovering the MSSA infections. More investigation is needed to further describe co-infections in patients with COVID-19 and to identify risk factors for the development of co-infections.
